# DNMT3A Cooperates with YAP/TAZ to Drive Gallbladder Cancer Metastasis

**DOI:** 10.1002/advs.202308531

**Published:** 2024-02-21

**Authors:** Sunwang Xu, Zhiqing Yuan, Cen Jiang, Wei Chen, Qiwei Li, Tao Chen

**Affiliations:** ^1^ Department of Biliary‐Pancreatic Surgery Renji Hospital, School of Medicine Shanghai Jiao Tong University Shanghai 200125 China; ^2^ Department of Thyroid and Breast Surgery The First Affiliated Hospital of Fujian Medical University Fuzhou 350005 China; ^3^ Central Laboratory Fujian Medical University Union Hospital Fuzhou 350001 China

**Keywords:** DNA methylation, DNMT3A, epithelial‐to‐mesenchymal transition, gallbladder cancer, metastasis, YAP

## Abstract

Gallbladder cancer (GBC) is an extremely lethal malignancy with aggressive behaviors, including liver or distant metastasis; however, the underlying mechanisms driving the metastasis of GBC remain poorly understood. In this study, it is found that DNA methyltransferase DNMT3A is highly expressed in GBC tumor tissues compared to matched adjacent normal tissues. Clinicopathological analysis shows that DNMT3A is positively correlated with liver metastasis and poor overall survival outcomes in patients with GBC. Functional analysis confirms that DNMT3A promotes the metastasis of GBC cells in a manner dependent on its DNA methyltransferase activity. Mechanistically, DNMT3A interacts with and is recruited by YAP/TAZ to recognize and access the CpG island within the CDH1 promoter and generates hypermethylation of the CDH1 promoter, which leads to transcriptional silencing of CDH1 and accelerated epithelial‐to‐mesenchymal transition. Using tissue microarrays, the association between the expression of DNMT3A, YAP/TAZ, and CDH1 is confirmed, which affects the metastatic ability of GBC. These results reveal a novel mechanism through which DNMT3A recruitment by YAP/TAZ guides DNA methylation to drive GBC metastasis and provide insights into the treatment of GBC metastasis by targeting the functional connection between DNMT3A and YAP/TAZ.

## Graphical abstract

Gallbladder cancer (GBC) is a lethal malignancy with highly aggressive behaviors, including liver metastasis or distant metastasis. Uncovering the mechanisms that drive GBC metastasis are desperately need. Here, the authors report a novel mechanism by which the DNA methyltransferase DNMT3A recruitment by YAP/TAZ guides DNA methylation to drive the metastasis of GBC and provides insights into the treatment for GBC metastasis.

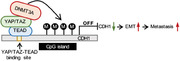

## 1 Introduction

Gallbladder cancer (GBC) is the most common type of biliary tract cancer and is a rare but highly lethal malignancy. Late diagnosis at an advanced or metastatic stage leads to an extremely poor prognosis, with a median overall survival of less than one year.^[^ ^1^ ^]^ GBC exhibits a highly aggressive behavior, and up to 70% of patients develop lymph node metastasis, liver metastasis, vascular invasion, perineural metastasis, or even distant metastasis, which limits the clinical therapeutic options owing to unresectable surgery and chemotherapy resistance.^[^ ^2^ ^]^ Metastasis is the leading cause of cancer‐related deaths. Therefore, identifying the key drivers of the high metastatic potential of GBC is necessary.

The development of aggressive phenotypes during GBC progression results from genetic and epigenetic dysregulation, including somatic mutations and amplifications, DNA methylation, and histone modifications.^[^ ^1b^ ^]^ DNA hypermethylation was more widely distributed than hypomethylation in GBC.^[^ ^3^ ^]^ Epigenetic alterations with hypermethylation in the promoter regions of tumor suppressor genes, such as TP53, CDKN2A, and RB1, have been found to drive tumorigenesis in GBC tissues.^[^ ^4^ ^]^ However, the molecular mechanisms underlying hypermethylation of these genes in GBC remain unclear.

DNA methylation occurs mainly at the fifth carbon of cytosine (5‐methylcytosine, 5mC) within CpG dinucleotides and is mediated by DNA methyltransferases (DNMTs) including DNMT1, DNMT3A, and DNMT3B. De novo DNA methylation is mainly catalyzed by DNMT3A and DNMT3B, but DNMT1 primarily maintains established DNA methylation during replication.^[^ ^5^ ^]^ In contrast, DNA methylation can be eliminated by DNA demethylase Ten‐eleven Translocation (TET) proteins. TET catalyzes the oxidation of 5mC to hydroxymethylated DNA (5‐hydroxymethylcytosine, 5hmC) and restores the active expression of target genes.^[^ ^6^ ^]^ Aberrant DNA methylation mediated by DNMTs contributes to alterations in the transcriptome and the deregulation of cellular pathways via epigenetic mechanisms. DNMT3A, with its methyltransferase activity, is required for tumorigenesis and the progression of various cancer types.^[^ ^7^ ^]^ Previously, we reported that DNMT3A could catalyze DNA methylation within the promoter regions of the elongator complex subunits to generate chemoresistance in GBC and could also be a therapeutic target to sensitize GBC to gemcitabine therapy,^[^ ^8^ ^]^ but its role in driving the metastasis of GBC remains unclear.

Here, we demonstrated that DNMT3A increases the aggressive behavior of GBC and drives GBC metastasis to the liver in a manner dependent on its DNA methyltransferase activity. We also report that DNMT3A can interact with YAP/TAZ, and that YAP/TAZ is required for recruiting DNMT3A to a specific genome, which has not yet been reported. These findings will be helpful in clarifying the metastasis‐driving functions of epigenetic alterations and in developing a novel therapeutic rationale for GBC metastasis.

## 2 Results

### 2.1 DNMT3A is Highly Expressed in GBC and Associated with Metastasis

**Figure 1:**
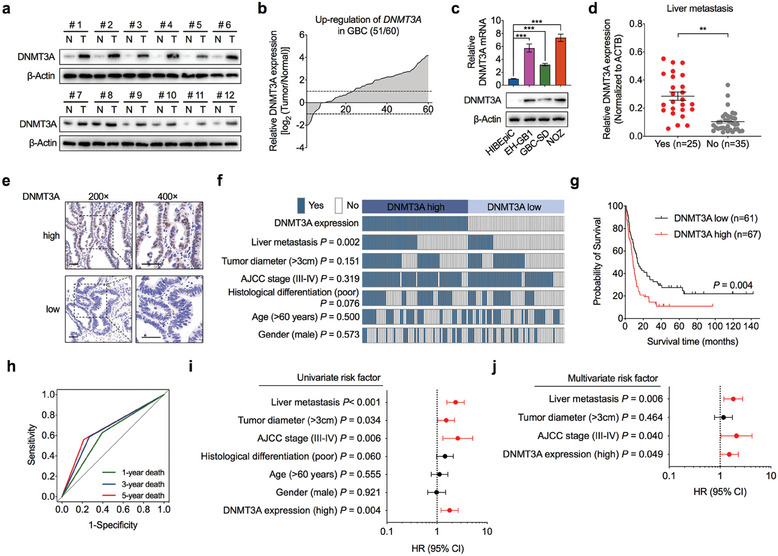
DNMT3A is highly expressed in GBC and associated with metastasis. a) The protein levels of DNMT3A in 12 representative paired GBC tumorous (T) tissues and adjacent normal tissues (N). b) Relative mRNA levels of DNMT3A in 60 cases of GBC tumorous tissues normalized to adjacent normal tissues. c) The mRNA (upper) and protein (lower) levels of DNMT3A in normal human intrahepatic biliary epithelial cell (HIBEpiC) and three GBC cell lines (EH‐GB1, GBC‐SD, and NOZ) (*n* = 3). Unpaired Student's *t* test, ^***^ *p*<0.001. d) Statistical analysis of DNMT3A mRNA levels in GBC tumorous tissues with (Yes, *n* = 25) and without (No, *n* = 35) liver metastasis. Unpaired Student's *t* test, ^**^ *p*<0.01. e) Representative images of immunohistochemistry (IHC) staining for DNMT3A protein in 128 cases of GBC tumorous tissues. Scale bar = 50 µm. f) Comparing the characteristics of liver metastasis, tumor diameter, AJCC stage, histological differentiation, age, and gender between GBC patients with DNMT3A high‐ (*n* = 67) and low‐expression (n = 61). Chi‐square test, the *P* value as indicated. g) Overall survival (OS) was compared between GBC patients with high and low expression of DNMT3A. Log‐rank test. h) Receiver operating characteristic (ROC) analysis for overall survivals was conducted based on the DNMT3A protein expression in GBC. Area under curve (AUC) of 0.616, 0.660, and 0.674, and *P* value of 0.024, 0.012, and 0.034 for 1‐year death, 3‐year death, and 5‐year death, respectively. i,j) Univariate (i) and multivariate (j) Cox regression analyses were performed in GBC patients. The bars correspond to 95% confidence intervals. HR, hazard ratio.

To determine the role of DNMT3A in GBC metastasis, we evaluated the expression of DNMT3A in fresh frozen tumor tissues derived from patients with GBC. DNMT3A expression was significantly upregulated in GBC tumor tissues compared to that in matched adjacent normal tissues at both protein and mRNA levels (**Figure** **1**a,b; Figure S1a, Supporting Information). In addition, both the mRNA and protein levels of DNMT3A were higher in the GBC cell lines than in the normal human intrahepatic biliary epithelial cell line (HIBEpiC) (Figure 1c; Figure S1b, Supporting Information). Moreover, we found that the level of DNMT3A in GBC tumor tissues was higher in patients with liver metastasis than in patients without liver metastasis (Figure 1d). Next, we assessed DNMT3A protein expression in a tissue microarray containing 128 paraformaldehyde‐fixed GBC tumor tissues using immunohistochemistry and evaluated the relationship between the expression level of DNMT3A and different clinicopathological characteristics in patients with GBC (Figure 1e). DNMT3A levels positively correlated with liver metastasis (Figure 1f), and high DNMT3A expression was strongly associated with shorter overall survival (OS) with GBC (Figure 1g). The 5‐year OS was substantially shorter in the DNMT3A‐high group than in the DNMT3A‐low group (Figure 1g). Receiver operating characteristic (ROC) curve analysis showed that DNMT3A expression predicted 1‐, 3‐, and 5‐year OS with GBC (Figure 1h). In addition, univariate and multivariate Cox regression analyses revealed that DNMT3A expression was an independent predictor of the clinical outcomes of patients with GBC, which was also comparable to liver metastasis and AJCC staging (Figure 1i,j). Together, these results confirmed that DNMT3A is pathologically and clinically associated with the aggressiveness and outcomes of patients with GBC.

### 2.2 DNMT3A Promotes GBC Metastasis in a Manner Dependent on its DNA methyltransferase activity

**Figure 2:**
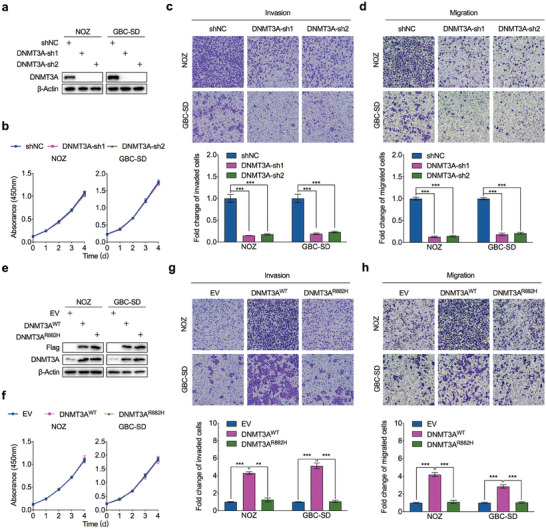
DNMT3A promotes the metastasis of GBC in vitro. a) Western blot was performed to detect DNMT3A expression in NOZ and GBC‐SD cells stably transfected with DNMT3A‐shRNAs (DNMT3A‐sh) or control shRNA (shNC). b) Cell viability assay by CCK‐8 method to compare the proliferation rate between DNMT3A‐depleted and control NOZ and GBC‐SD cells (*n* = 3). c,d) Representative images (upper) and statistical bar graphs (lower) depicting the relative cell invasion rate (c) and migration rate (d) in NOZ and GBC‐SD cells with or without DNMT3A deletion (*n* = 3). e) Western blot was performed to detect ectopic expressed DNMT3A levels in NOZ and GBC‐SD cells stably transfected with DNMT3A wild‐type construct (DNMT3A^WT^), catalytic mutation construct (DNMT3A^R882H^), or empty vector (EV). f) Cell viability assay by CCK‐8 method to compare the proliferation rate between DNMT3A^WT^, DNMT3A^R882H^, and EV stably expressed NOZ and GBC‐SD cells (*n* = 3). g,h) Representative images (upper) and statistical bar graphs (lower) depicting the relative cell invasion rate (g) and migration rate (h) in NOZ and GBC‐SD cells with DNMT3A^WT^, DNMT3A^R882H^, or EV stably expressed NOZ and GBC‐SD cells (*n* = 3). Unpaired Student's *t* test in (c,d,g,h), ^**^ *p*<0.01, ^***^ *p*<0.001.

Given the hypothesis that DNMT3A drives the liver metastasis of GBC, we tested the biological function of DNMT3A in GBC cells in vitro and in vivo. Upon silencing DNMT3A expression using two independent shRNAs in two GBC cell lines with different DNMT3A expression levels, NOZ and GBC‐SD, no proliferative changes were observed in DNMT3A‐depleted cells (**Figure** **2**a,b; Figure S2a, Supporting Information). As expected, the invasive and migratory capacities of both GBC cell lines were markedly inhibited by DNMT3A depletion (Figure 2c,d). We then overexpressed DNMT3A in both GBC cell lines to test whether the inhibitory effects on aggressiveness of GBC cells resulted from the specific knockdown of DNMT3A. The results showed that DNMT3A overexpression enhanced the invasion and migration abilities of GBC cells but had no effect on cell proliferation (Figure 2e–h; Figure S2b, Supporting Information). These data indicated that DNMT3A promotes GBC metastasis in a cell proliferation‐independent manner.

The loss‐of‐function mutation at the arginine 882 residue (R882) of DNMT3A inactivates its methyltransferase activity and generates hypomethylation at specific CpG sites in the genome.^[^ ^9^ ^]^ To examine whether the methyltransferase activity of DNMT3A was necessary for the aggressive phenotype of GBC cells, we ectopically expressed a DNMT3A catalytic mutant construct (R882H) in GBC cells (Figure 2e). As shown in Figure 2g,h, catalytically inactivated DNMT3A did not increase the invasive and migratory capacities of GBC cells.

**Figure 3:**
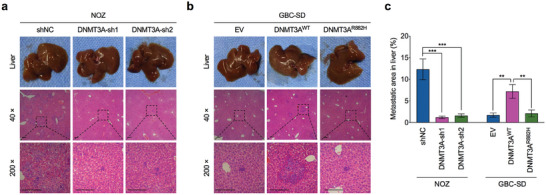
DNMT3A promotes the metastasis of GBC in vivo. a) Representative images of liver metastasis nodules (upper) and H&E staining (middle and lower) in DNMT3A‐depleted and control NOZ cells (*n* = 6). b) Representative images of liver metastasis nodules (upper) and H&E staining (middle and lower) in DNMT3A wild‐type construct (DNMT3A^WT^), catalytic mutation construct (DNMT3A^R882H^), or empty vector (EV) ectopic expressed GBC‐SD cells (*n* = 6). c) Statistical bar graphs for liver metastatic area in (a) and (b) (*n* = 6). Unpaired Student's *t* test in (c), ^**^ *p*<0.01, ^***^ *p*<0.001.

Consistent with the finding that DNMT3A induces an aggressive phenotype of GBC in vitro, the in vivo tumor xenograft assay indicated that silencing DNMT3A inhibited GBC cell metastasis to the liver (**Figure** **3**a,c). In contrast, the overexpression of the catalytically active wild‐type DNMT3A promoted the development of liver metastasis in GBC cells, but the catalytically disabled mutant DNMT3A failed (Figure 3b,c). Collectively, these data indicated that DNMT3A and its DNA methyltransferase activity were required for the metastasis of GBC cells.

### 2.3 DNMT3A Catalyzes the CpG Methylation of CDH1 Promoter to Drive EMT

**Figure 4:**
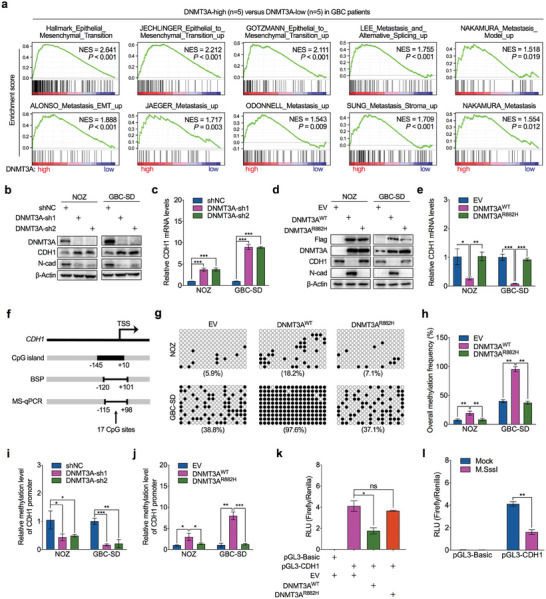
DNMT3A catalyzes the DNA methylation of CDH1 promoter to drive EMT. a) Gene Set Enrichment Analysis (GSEA) was conducted to show the enriched gene signatures in DNMT3‐high expressed GBC tissues. b) Western blot was performed to detect protein levels of the epithelial marker CDH1 and mesenchymal marker N‐cadherin (N‐Cad) in DNMT3A‐depleted and control NOZ and GBC‐SD cells. c) RT‐qPCR was performed to detect mRNA levels of CDH1 in DNMT3A‐depleted and control NOZ and GBC‐SD cells (*n* = 3). d) Western blot was performed to detect protein levels of CDH1 and N‐cadherin in DNMT3A wild‐type construct (DNMT3A^WT^), catalytic mutation construct (DNMT3A^R882H^), or empty vector (EV) ectopic expressed NOZ and GBC‐SD cells. e) RT‐qPCR was performed to detect mRNA levels of CDH1 in DNMT3A wild‐type construct, catalytic mutation construct, or empty vector stably expressed NOZ and GBC‐SD cells (*n* = 3). f) Schematic diagram of CpG island on CDH1 gene promoter region around the transcription start site (TSS). g) Bisulfite sequencing PCR (BSP) assay was performed to analyze the methylation status of CDH1 promoter in NOZ and GBC‐SD cells with DNMT3A wild‐type construct, catalytic mutation construct, or empty vector stably expressed. 17 individual CpG sites within the CGI were sequenced. Open circle indicates unmethylated CpG site and filled circle indicates methylated CpG site. h) The bar graph depicted the overall methylation rate of CDH1 promoter in (g) (*n* = 3). i) MS‐qPCR was performed to determine the methylation levels of CDH1 promoter in DNMT3A‐depleted and control NOZ and GBC‐SD cells (*n* = 3). j) MS‐qPCR was performed to determine the methylation levels of CDH1 promoter in DNMT3A wild‐type construct, catalytic mutation construct, or empty vector stably expressed NOZ and GBC‐SD cells (*n* = 3). k) HEK293T cells were transfected with CDH1 promoter construct together with DNMT3A wild‐type construct, catalytic mutation construct, or empty vector stably, and luciferase assay was conducted to evaluate CDH1 promoter activities. RLU, relative light units (*n* = 3). l) HEK293T cells were transfected with in vitro M.SssI or mock methylated CDH1 promoter and the promoter activity was compared by luciferase assay (*n* = 3). Unpaired Student's *t* test in (c,e,h–l), ^*^ *p*<0.05, ^**^ *p*<0.01, ^***^ *p*<0.001.

To investigate whether the above results generated by DNMT3A are related to the aggressive signatures of GBC, we performed the Gene Set Enrichment Analysis (GSEA) of the transcriptome of GBC tumor tissues from our published GBC transcriptional profiles (GSE139682).^[^ ^8a^ ^]^ GSEA results showed that gene signatures representing metastasis, especially epithelial‐to‐mesenchymal transition (EMT), were significantly enriched in patients with higher levels of DNMT3A expression than in those with lower expression of this gene (**Figure** **4**a). To confirm the effects of DNMT3A on EMT in GBC, we assessed the expression of EMT markers in GBC cells in which DNMT3A was silenced or overexpressed. DNMT3A deficiency restored the expression of the epithelial marker CDH1 (encoding E‐cadherin protein) but decreased the expression of the mesenchymal marker N‐cadherin (Figure 4b,c; Figure S3a, Supporting Information). Notably, the expression of CDH1 was inhibited and that of N‐cadherin was increased by ectopically expressed wild‐type DNMT3A in GBC cells. However, the expression of these genes was minimally altered by the catalytically disabled mutant DNMT3A (Figure 4d,e; Figure S3b, Supporting Information), strongly suggesting that DNMT3A promotes EMT via its methyltransferase activity.

Next, we attempted to identify the mechanisms through which DNMT3A inhibits the transcription of CDH1 to determine its promoting effects on EMT. Using the MethPrimer webserver,^[^ ^10^ ^]^ a CpG island (CGI) containing 17 CpG sites between −145 to +10 bp around the transcription start site (TSS) of the CDH1 proximal promoter region was identified (Figure 4f). To investigate whether DNMT3A can methylate CGI on the CDH1 promoter in GBC cells, we used a bisulfite sequencing PCR (BSP) assay to visualize the methylation status of CGI on the CDH1 promoter. Compared to empty vector‐ or R882H mutant DNMT3A‐transfected GBC cells, wild‐type DNMT3A‐overexpressing cells had a markedly higher overall methylation percentage in the CGI of the CDH1 promoter (Figure 4g,h). This result was validated using methylation‐specific quantitative PCR (MS‐qPCR). The methylation level of CGI in the CDH1 promoter was decreased by DNMT3A silencing or increased by overexpression of catalytically active wild‐type DNMT3A (Figure 4i,j). Luciferase assays showed that the DNMT3A methyltransferase activity‐induced methylation of CGI in the CDH1 promoter repressed CDH1 transcription (Figure 4k). As confirmed by in vitro methylation of the CDH1 promoter, its transcriptional activity was inhibited (Figure 4l), suggesting that DNA methylation directly represses CDH1 promoter activity in vitro. Given these findings, we conclude that DNMT3A drives the EMT process in GBC cells by catalyzing hypermethylation of the CDH1 promoter to silence the expression of CDH1.

### 2.4 DNMT3A Interacts with YAP/TAZ

**Figure 5:**
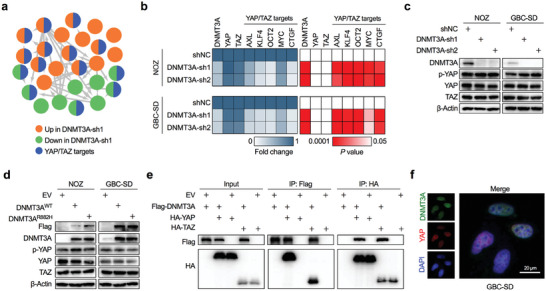
DNMT3A interacts with YAP/TAZ. a) A PCR array based analysis for tumor metastasis related genes indicated that YAP/TAZ transcriptional targets were differently expressed in DNMT3A‐depleted and control NOZ cells. b) RT‐qPCR was performed to validate the representative differentially expressed YAP/TAZ target genes in DNMT3A‐depleted and control NOZ and GBC‐SD cells (*n* = 3). Unpaired Student's *t* test, the *P* value as indicated. c) Western blot was performed to detect protein levels of the YAP, Ser127‐phosphorylated YAP (p‐YAP), and TAZ in DNMT3A‐depleted and control NOZ and GBC‐SD cells. d) Western blot was performed to detect protein levels of the YAP, p‐YAP, and TAZ in DNMT3A wild‐type construct, catalytic mutation construct, or empty vector stably expressed NOZ and GBC‐SD cells. e) HEK293T cells were transfected with empty vector, Flag‐DNMT3A, HA‐YAP, HA‐TAZ alone or together as indicated, and co‐immunoprecipitation (Co‐IP) was conducted to identify the interaction between DNMT3A and YAP/TAZ. f) Immunofluorescence staining of DNMT3A (green) and YAP (red) in wild‐type GBC‐SD cells. Scale bar = 20 µm.

To decipher how DNMT3A recognizes CGI on the CDH1 promoter in GBC cells, we analyzed GSEA results from our published GBC transcriptional profile (GSE139682) and found that the evolutionarily conserved signature of the Hippo pathway effector YAP (Yes‐associated protein) was positively correlated with DNMT3A expression in GBC (Figure S4a, Supporting Information). To identify the correlation between DNMT3A and YAP, we performed tumor metastasis‐associated gene expression profiling in DNMT3A‐depleted and control GBC cells using a PCR array. The results showed that the most dysregulated genes altered by DNMT3A depletion were downstream targets of the co‐transcription factor YAP/TAZ (**Figure** **5**a; Figure S4b, Supporting Information), and their expression changes in DNMT3A‐depleted cells were highly consistent with changes in YAP depletion in GBC cells (Figure S4b–d, Supporting Information). As validated by RT‐qPCR assays, DNMT3A deficiency severely decreased the expression of YAP/TAZ transcriptional targets (Figure 5b). Hippo pathway and particularly its downstream effectors YAP and TAZ are essential for tumor initiation and malignant progression.^[^ ^11^ ^]^ Our results suggest that DNMT3A cooperates with YAP/TAZ to generate aggressive behavior in GBC.

The Hippo pathway negatively regulates YAP/TAZ activity via a kinase cascade to phosphorylate YAP/TAZ, resulting in its cytoplasmic localization, ubiquitination, and degradation. When Hippo signaling is turned off, YAP/TAZ accumulates in the nucleus and activates the transcription of specific genes to induce cancer cell proliferation, survival, mobility, and metastasis.^[^ ^12^ ^]^ Therefore, we examined whether the association between DNMT3A and YAP/TAZ is linked to the Hippo pathway‐controlled pathway. First, we assayed YAP with its phosphorylation and also TAZ protein levels in GBC cells transfected with DNMT3A shRNA or ectopically expressed constructs. However, neither DNMT3A depletion nor DNMT3A overexpression changed the expression of YAP and TAZ, and no changes were observed in the phosphorylation of YAP at serine 127, a target site for the Hippo pathway downstream kinase (Figure 5c,d; Figure S5a,b, Supporting Information). Next, we performed an immunoprecipitation assay and found that DNMT3A physically interacted with YAP/TAZ (Figure 5e). Moreover, DNMT3A co‐localized with YAP in the nucleus of GBC cells (Figure 5f). In conclusion, YAP/TAZ may serve as a key guide for DNMT3A recruitment to specific genomic locations in a Hippo pathway‐independent manner.

### 2.5 YAP/TAZ Induces the Metastasis of GBC

**Figure 6:**
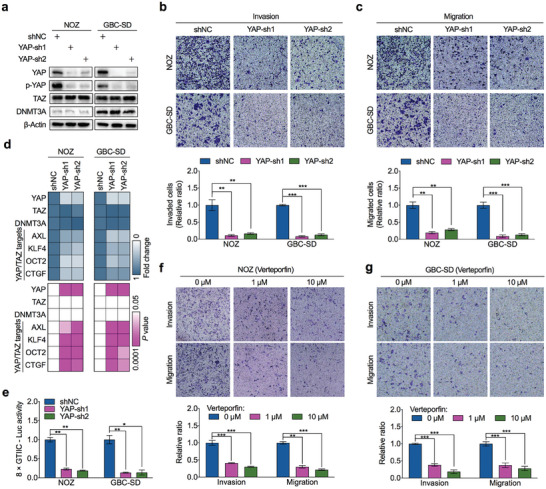
YAP/TAZ promotes the metastasis of GBC. a) Western blot was performed to detect YAP, p‐YAP, TAZ and DNMT3A expression in NOZ and GBC‐SD cells stably transfected with YAP‐shRNAs (YAP‐sh) or control shRNA (shNC). b,c) Representative images (upper) and statistical bar graphs (lower) depicting the relative cell invasion rate (b) and migration rate (c) in NOZ and GBC‐SD cells with or without YAP deletion (*n* = 3). d) RT‐qPCR was performed to detect the expression of YAP/TAZ target genes in YAP‐depleted and control NOZ and GBC‐SD cells (*n* = 3). e) YAP‐depleted and control NOZ or GBC‐SD cells were transfected with 8×GTIIC‐Luc constructs, and the luciferase assay was performed to verify the effect of YAP deletion on 8×GTIIC‐Luc activity in GBC cells (*n* = 3). f,g) Representative images for depicting the cell invasion and migration in wild‐type NOZ f) and GBC‐SD g) cells treated with concentration‐gradient of YAP inhibitor verteporfin (*n* = 3). Unpaired Student's *t* test in b–g), ^*^ *p*<0.05, ^**^ *p*<0.01, ^***^ *p*<0.001.

Although YAP/TAZ is required for metastasis in various solid tumors,^[^ ^13^ ^]^ and the oncogenic role of YAP in the tumor growth of GBC has been confirmed,^[^ ^14^ ^]^ the biological function of YAP/TAZ in driving GBC metastasis remains uncertain. We verified the biological validity of YAP/TAZ in GBC metastasis. By knocking down YAP in GBC cells with two independent shRNAs, we found that YAP deletion did not interfere with DNMT3A expression (**Figure** **6**a; Figure S6a, Supporting Information). Similar to the finding that DNMT3A silencing mediated decreased invasion and migration of GBC cells, the invasion and migration capacities of GBC cells were reduced by YAP deletion (Figure 6b,c). Biologically, the transcription of YAP/TAZ target genes was significantly decreased following YAP deletion (Figure 6d). To read out of YAP/TAZ transcriptional activity on driving GBC metastasis more directly, we utilized the 8×GTIIC‐Luc reporter, a synthetic and well‐characterized YAP/TAZ‐responsive luciferase reporter for evaluating YAP/TAZ activity.^[^ ^15^ ^]^ As expected, YAP deletion repressed the 8×GTIIC‐Luc reporter activity in GBC cells (Figure 6e). Together, these results rule out the possibility that the YAP‐mediated dysregulation of aggressive phenotypes in GBC cells is dependent on YAP/TAZ transcriptional activity.

Additionally, we used verteporfin, an inhibitor of YAP/TAZ that interferes with YAP/TAZ binding to chromatin, to inhibit the transcriptional activity of YAP/TAZ in GBC cells. As shown in Figure 6f,g, pharmacological inhibition of YAP/TAZ by verteporfin decreased the invasion and migration of GBC cells in a dose‐dependent manner. These data further supported the oncogenic role of YAP/TAZ in activating metastasis for aggressive GBC cells.

### 2.6 YAP/TAZ is Required for DNMT3A Recognition of the CDH1 Promoter

**Figure 7:**
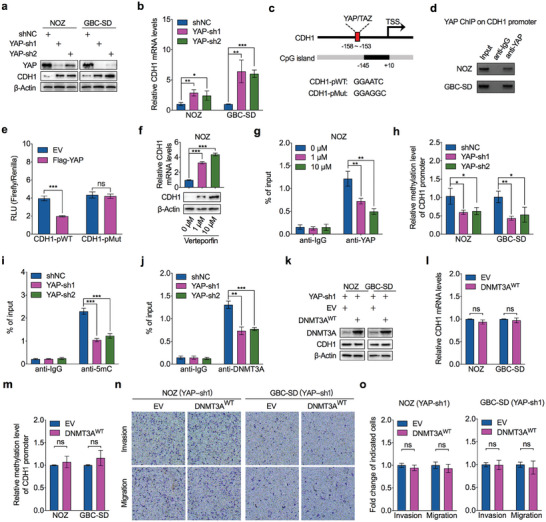
YAP/TAZ recruits DNMT3A to methylate CDH1 promoter. a) Western blot was performed to detect protein level of CDH1 in YAP‐depleted and control NOZ and GBC‐SD cells. b) RT‐qPCR was performed to detect mRNA levels of CDH1 in YAP‐depleted and control NOZ and GBC‐SD cells (*n* = 3). c) Schematic diagram of the luciferase reporter constructs containing the promoter of CDH1 with conserved wild‐type (CDH1‐pWT) or mutant (CDH1‐pMut) YAP/TAZ‐TEAD binding site. d) ChIP‐PCR was performed to detect the YAP enrichment at the YAP/TAZ‐TEAD binding site contained region of CDH1 promoter. e) HEK293T cells were transfected with wild‐type or mutant CDH1 promoter constructs together with YAP overexpression or empty vector, and luciferase assay was performed to analyze the wild‐type and mutant CDH1 promoter activity (*n* = 3). f) RT‐qPCR and western blot was performed to detect CDH1 mRNA (upper) and protein (lower) levels in NOZ cells treated with concentration‐gradient of verteporfin for 24 h (*n* = 3). g) ChIP‐qPCR was conducted to evaluate the enrichment of YAP on CDH1 promoter with or without verteporfin treatment for 24 h in NOZ cells (*n* = 3). h) MS‐qPCR was performed to determine the methylation levels of CDH1 promoter in YAP‐depleted and control NOZ and GBC‐SD cells (*n* = 3). i) ChIP‐qPCR was performed to evaluate of 5mC occupancy at CGI of CDH1 promoter region in YAP‐depleted or control NOZ cells (n = 3). j) ChIP‐qPCR analysis for DNMT3A occupancy at CGI of CDH1 promoter region in YAP‐depleted or control NOZ cells (*n* = 3). k) Western blot was performed to detect CDH1 protein levels in YAP‐depleted NOZ and GBC‐SD cells transfected with DNMT3A overexpression constructs or empty vector. l) RT‐qPCR was performed to detect CDH1 mRNA levels in YAP‐depleted NOZ and GBC‐SD cells transfected with DNMT3A overexpression constructs or empty vector (*n* = 3). m) MS‐qPCR was performed to determine the methylation levels of CDH1 promoter in YAP‐depleted NOZ and GBC‐SD cells transfected with DNMT3A overexpression constructs or empty vector (*n* = 3). n,o) Representative images (n) and statistical bar graphs (o) depict the relative cell invasion and migration rate in YAP‐depleted NOZ and GBC‐SD cells transfected with DNMT3A overexpression constructs or empty vector (*n* = 3). Unpaired Student's *t* test in b,e–j,l,m,o), ^*^ *p*<0.05, ^**^ *p*<0.01, ^***^ *p*<0.001, ns, not significant.

To elucidate the functional relationship between DNMT3A and YAP/TAZ in EMT of GBC, we examined the role of YAP in EMT. As shown in **Figure** **7**a,b (Figure S7a, Supporting Information), YAP silencing restored CDH1 expression at both the protein and mRNA levels, suggesting that YAP/TAZ negatively regulated CDH1 transcription to drive EMT. YAP/TAZ have no direct DNA‐binding capacity but need to interact with members of the TEAD family, which are important mediators of the oncogenic properties of YAP/TAZ.^[^ ^16^ ^]^ Therefore, we analyzed the promoter sequence of CDH1 to determine the YAP/TAZ‐TEAD binding sites. A putative site with a consensus sequence GGAATC for YAP/TAZ‐TEAD binding, located upstream of the CGI in the CDH1 promoter, was found (Figure 7c). As confirmed using the ChIP‐PCR assay, YAP bound to the YAP/TAZ‐TEAD binding site contained in a region on the CDH1 promoter (Figure 7d). To further validate whether the YAP/TAZ‐TEAD binding site on the CDH1 promoter is required for YAP function, we introduced a site‐specific mutation within the YAP/TAZ‐TEAD binding site, which caused the CDH1 promoter construct to impede YAP/TAZ‐TEAD access (Figure 7c). A luciferase assay confirmed that this mutation interfered with the ability of YAP to repress CDH1 promoter activity (Figure 7e). Remarkably, we found that treatment with the YAP/TAZ inhibitor verteporfin increased the expression of CDH1 at transcriptional levels (Figure 7f; Figure S7b, Supporting Information), by impeding YAP binding to the YAP/TAZ‐TEAD site on the CDH1 promoter in a dose‐dependent manner (Figure 7g). These results indicated that YAP/TAZ represses CDH1 transcription by accessing the YAP/TAZ‐TEAD‐binding site in the CDH1 promoter.

The YAP/TAZ‐TEAD binding site is close to the CGI of the CDH1 promoter. Engaging with the coregulated genes for YAP and DNMT3A, we hypothesized that YAP could cooperate with DNMT3A‐mediated DNA methylation or YAP is required for DNMT3A catalyzing the promoter methylation to inhibit CDH1 transcriptional activity. To test this hypothesis, we performed MS‐qPCR to determine the methylation status of CDH1 promoter in YAP‐depleted and control GBC cells. YAP depletion interfered with CGI methylation of the CDH1 promoter (Figure 7h). Notably, ChIP‐qPCR analysis confirmed that YAP depletion reduced 5mC occupancy within the CDH1 promoter (Figure 7i) and prevented DNMT3A from binding to the CDH1 promoter (Figure 7j), suggesting that YAP was required for DNMT3A to bind to chromatin and catalyze DNA methylation. To further validate this finding, we restored DNMT3A expression in YAP‐depleted cells (Figure 7k). Interestingly, ectopically expressed DNMT3A failed to repress CDH1 expression in the absence of endogenous YAP expression (Figure 7k,l; Figure S7c, Supporting Information), and the methylation status of the CDH1 promoter was not altered by DNMT3A in YAP‐depleted cells (Figure 7m). More importantly, ectopically expressed DNMT3A did not restore the impaired invasion and migration capabilities of YAP‐depleted GBC cells (Figure 7n,o). Taken together, these data demonstrate that DNMT3A and YAP/TAZ can functionally interact to repress CDH1 transcription through YAP/TAZ recruitment of DNMT3A binding to the CDH1 promoter and catalyzing the hypermethylation of CGI in the CDH1 promoter and that YAP/TAZ is required for DNMT3A recognition of the specific CGI on the promoter.

### 2.7 Clinical Relevance of DNMT3A, YAP, and CDH1 in GBC

**Figure 8:**
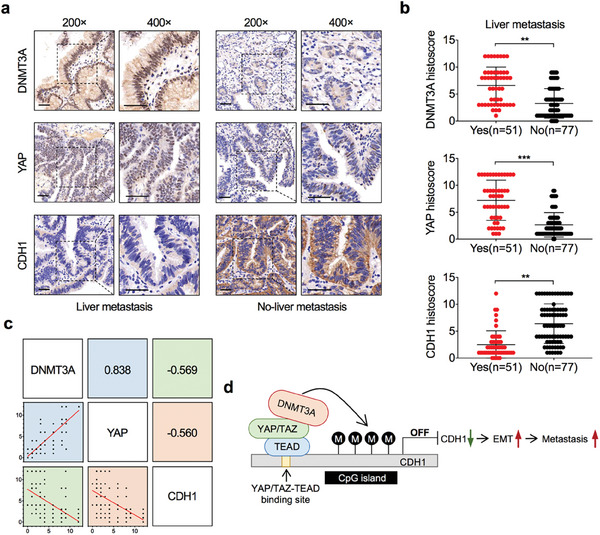
Clinical relevance of DNMT3A, YAP, and CDH1 in GBC. a) Representative immunohistochemistry of DNMT3A (upper), YAP (middle), and CDH1 (lower) proteins in GBC tissues from patients with (n = 51) or without (*n* = 77) liver metastasis. Scale bar = 50 µm. b) Statistical analysis of the histoscore of DNMT3A (upper), YAP (middle), and CDH1 (lower) proteins in GBC tissues with (Yes, *n* = 51) or without (No, *n* = 77) liver metastasis. Unpaired Student's *t* test, ^**^ *p*<0.01, ^***^ *p*<0.001. c) Correlations among DNMT3A, YAP, and CDH1 levels in GBC tissues (*n* = 128). Pearson correlation coefficient test. d) Schematic diagram for DNMT3A cooperates with YAP/TAZ to drive the metastasis of GBC.

We validated the translational and clinical relevance of functional DNMT3A and YAP/TAZ interactions and their co‐downstream targets in GBC tissues. Using immunohistochemical staining, we evaluated the protein expression of DNMT3A, YAP, and CDH1 in GBC tissues with and without liver metastasis. The results showed that GBC tissues from patients with liver metastasis had higher DNMT3A and YAP expression but lower CDH1 expression than those from patients without liver metastasis (**Figure** **8**a,b). Overall, the expression level of DNMT3A was positively correlated with YAP but negatively correlated with CDH1 (Figure 8c). YAP expression also negatively correlated with CDH1 expression (Figure 8c).

Altogether, our findings show that YAP/TAZ is required for DNMT3A to recognize, access, and hypermethylate the CDH1 promoter, resulting in an accelerated EMT process that drives GBC metastasis (Figure 8d).

## 3 Discussion

Here, we described a direct functional link between DNMT3A and the transcriptional coactivator YAP/TAZ to control GBC metastasis. Mechanistically, DNMT3A binds to and methylates the CDH1 promoter in a manner dependent on the recruitment of YAP/TAZ via a physical interaction. To the best of our knowledge, this is the first study to investigate the interaction between DNMT3A and YAP/TAZ. Our analyses of clinical data and patient characteristics provided evidence for the relevance of our finding that DNMT3A and its methyltransferase activity drive GBC metastasis.

DNMT3A‐mediated de novo DNA hypermethylation plays an important role in regulating multiple biological processes, including embryonic development, somatic cell reprogramming, and even oncogenesis.^[^ ^17^ ^]^ Although DNMT3A and its genetic mutations are well known in hematological malignancies,^[^ ^18^ ^]^ the potential roles of DNMTA in solid tumors, especially in tumor aggressiveness, remain largely unknown. The most well‐known biochemical function of DNMT3A is to catalyze DNA methylation, resulting in the silencing of genes including CDH1.^[^ ^7^, ^19^ ^]^ However, the silencing of CDH1 expression by DNMT3A, how DNMT3A recognizes CGI on the CDH1 promoter, and whether this is specific to the methyltransferase activity of DNMT3A lack direct evidence. Here, we provide evidence supporting the catalytic‐activity‐dependent role of DNMT3A in driving GBC metastasis, which is strongly correlated with DNA methylation‐mediated CDH1 transcriptional silencing, as verified by the introduction of a catalytically disabled mutation into DNMT3A. The R882H hotspot mutation located in the MTase domain of DNMT3A is a loss‐of‐function mutation that impairs the CpG DNA methyltransferase enzymatic activity of DNMT3A.^[^ ^9^, ^20^ ^]^ The R882H mutation also has a dominant‐negative effect on DNMT3A by forming wild‐type/R882H heterocomplex which affects wild‐type DNMT3A tetramerization.^[^ ^9^, ^21^ ^]^ However, the heterocomplex formation of DNMT3A wild‐type and R882H mutants may not always induce a dominant‐negative effect in human cells^[^ ^22^ ^]^ because the R882H mutant generates a dominant‐negative effect in a dose‐dependent manner, inhibiting the methyltransferase activity of wild‐type DNMT3A.^[^ ^23^ ^]^ In the present study, we showed that the R882H mutation impairs the DNMT3A‐mediated hypermethylation of CGI within the CDH1 promoter and reduces the ability of DNMT3A to promote the invasion and migration of GBC cells. However, we did not observe a dominant‐negative effect of the R882H mutant on the inhibition of DNA methylation of the CDH1 promoter or the metastasis of GBC. We speculate that this phenomenon may be because our lentivirus‐delivered overexpression system was unable to overexpress the R882H mutant to a sufficiently high protein level, which achieves the dominant‐negative effects of the wild‐type/R882H heterocomplex but only exhibits the enzymatic loss of functional activity of DNMT3A in GBC cells. Overall, we provide convincing evidence that the DNA methylation activity of DNMT3A is required for CDH1 silencing and GBC aggressiveness.

DNMT3A can interact with chromatin‐remodeling proteins or engage in crosstalk with chromatin or histone modifications to gain access to preferred genomic loci that regulate specific gene transcription.^[^ ^24^ ^]^ For instance, NSD1‐mediated methylation of H3K36me2 or PRC1‐mediated H2AK119ub can recruit DNMT3A to intergenic genomes,^[^ ^25^ ^]^ but CEBPA or SALL3 can physically interact with DNMT3A to prevent DNMT3A access to DNA substrates.^[^ ^26^ ^]^ However, the mechanism through which DNMT3A is recruited to its target genome remains unknown. By interpreting the finding that the loss of YAP disrupts DNMT3A binding to the CDH1 promoter and eliminates DNMT3A‐catalyzed DNA methylation in the CDH1 promoter, we verified the possibility that YAP/TAZ activity is critical for DNMT3A recognition of a specific gene promoter. Our results provide evidence for a previously unrecognized mechanism by which DNMT3A binds to a specific genomic region in a YAP/TAZ‐dependent manner. In addition to CDH1, we found that a subset of metastasis‐related genes could be co‐regulated by DNMT3A and YAP. However, it remains unclear whether these genes share YAP/TAZ‐TEAD binding sites and CpG methylation elements in the promoter region for the recruitment of YAP/TAZ and DNMT3A, which requires further validation. Further studies are needed to determine whether YAP/TAZ recruitment is required for the genome‐wide recognition of DNMT3A.

Although YAP is known to contribute to DNA methylation remodeling, in which YAP depletion generates hypomethylation of the genome,^[^ ^27^ ^]^ the underlying molecular mechanism remains uncertain. The YAP/TAZ‐TEAD complex engages with histone‐modifying enzymes, such as the PRC2 member EZH2, multifunction transcription factor YY1, and EMT transcription factor SLUG, for YAP‐mediated transcriptional repression.^[^ ^28^ ^]^ Our findings reveal a novel functional link between DNA methyltransferases and YAP. This finding is supported by the physical interaction between DNMT3A and YAP/TAZ, and the YAP‐dependent recruitment of DNMT3A to the genome. A recent study found that the 5‐methylcytosine dioxygenase TET1 interacts with TEAD to cause regional DNA demethylation in YAP target genes, facilitating transcriptional activation in hepatomegaly and tumorigenesis.^[^ ^29^ ^]^ Based on our findings, YAP/TAZ cooperates with DNA methylation to facilitate tumor malignancy in two ways: by interacting with the DNA methyltransferase DNMT3A to induce DNA methylation‐mediated transcriptional repression of tumor suppressor genes, and by interacting with the DNA demethylase TET1 to eliminate DNA methylation and activate oncogenes.

Interestingly, YAP promotes the proliferation of GBC,^[^ ^14^ ^]^ but we did not find any influence of DNMT3A on GBC growth. YAP and TAZ are the key downstream effectors of the Hippo pathway. Kinases in the Hippo pathway phosphorylate YAP at serine 127, resulting in the cytoplasmic sequestration and protein degradation of YAP and limiting its coactivating transcription function. Deregulation of the Hippo pathway results in the dephosphorylation and nuclear localization of YAP to control the expression of cell cycle regulators and accelerate the growth of tumor cells.^[^ ^30^ ^]^ In the present study, we showed that DNMT3A interacts with YAP/TAZ but does not modulate YAP/TAZ expression or cytoplasmic sequestration, revealing a Hippo pathway‐independent role for YAP in recruiting DNMT3A access to specific genome locations. In addition, our findings regarding the inconsistent function of DNMT3A and YAP in GBC growth led us to speculate that YAP‐induced expression of cell cycle regulators might not rely on DNMT3A‐mediated DNA methylation, which needs to be further explored.

Drugs that modify DNA methylation status have been evaluated as potential therapeutic agents for certain cancer types in preclinical and clinical studies, and some have already been approved for treating hematological cancers.^[^ ^31^ ^]^ For example, 5‐2′‐deoxycytidine (decitabine), a DNA methyltransferase inhibitor, has been approved by the US Food and Drug Administration to treat myelodysplastic syndromes and chronic myelomonocytic leukemia.^[^ ^32^ ^]^ The therapeutic efficacy of DNA demethylating agents in solid tumors remains unclear. Previously, using in vitro and in vivo models, we demonstrated that targeting DNA methylation is an effective therapeutic strategy to overcome chemoresistance in GBC cells.^[^ ^8b^ ^]^ In this study, we found that blocking the function of YAP/TAZ with an inhibitor specifically reduced the invasive and migratory abilities of GBC cells. Thus, we speculate whether combining a DNA methylation inhibitor with a YAP/TAZ inhibitor could be an efficient strategy for preventing GBC metastasis. However, this requires further investigation.

In summary, by elucidating a novel functional link between YAP/TAZ and DNMT3A‐mediated DNA methylation during cancer metastasis, we identified a previously unknown interaction between DNMT3A and YAP/TAZ. We also uncovered the mechanisms through which DNMT3A, and its methyltransferase activity generate hypermethylation of CDH1 to facilitate GBC metastasis. These mechanistic findings may explain how DNMT3A, and YAP/TAZ play oncogenic roles in aggressive phenotypes of patients with GBC.

## 4 Experimental Section

### Cell Culture and Reagents

Human GBC cell line NOZ was purchased from the Health Science Research Resources Bank (Osaka, Japan), GBC‐SD cell was purchased the Cell Bank of Type Culture Collection of Chinese Academy of Sciences, and human embryonic kidney 293 T (HEK293T) cells were purchased from the American Type Culture Collection, and cultured in William's E medium, DMEM, and DMEM (Hyclone), which was supplemented with 10% fetal bovine serum (FBS) (Gibco) in a humidified atmosphere of 5% CO_2_ at 37 °C. All cell cultures were ensured to be mycoplasma‐negative cultures by monthly mycoplasma tests. Verteporfin, and puromycin were purchased from MedChemExpress, polyethylenimine was purchased from Polysciences, and polybrene was purchased from (Yeasen)

### Cell Transfection

DNMT3A and YAP targeting short harpin RNA (shRNA) and non‐specific control shRNA (shNC) used in this study were obtained from Biochemistry and Molecular Cell Biology, Shanghai Jiao Tong University School of Medicine. The coding sequence of DNMT3A was cloned into the pCDH‐CMV‐MCS‐EF1‐Puro vector. For lentivirus production and infection, HEK293T cells with 80%–90% confluency in 10‐mm dishes were co‐transfected with 4.4 µg of the required knockdown or overexpressed plasmid, 3.3 µg of psPAX and 2.2 µg of pMD2.G with 30 µl of polyethylenimine. After transfection for 6 h at 37 °C, the medium was replaced, and the lentivirus‐containing medium was harvest 72 h later. Then the GBC cells were infected by the lentivirus supplemented with polybrene for 24 h, and the puromycin‐resistant cells were harvest as the stable transfected cell lines. The sense sequence of shRNAs were: DNMT3A‐sh1, 5′‐CCCAAGGTCAAGGAGATTA‐3′; DNMT3A‐sh2, 5′‐CGGCTCTTCTTTGAGTTCT‐3′; YAP‐sh1, 5′‐AGGTGATACTATCAACCAA‐3′; YAP‐sh2, 5′‐AGCTCAGATCCTTTCCTTA‐3′.

### Migration and Invasion Assay

For the migration assay, the GBC cells were starved without FBS for 4 h, then resuspended with a concentration of 20 000 cells in 100 µl culture medium without FBS, and cultured in the upper chamber of non‐coated transwell inserts in the 12‐well plate (Corning). 500 µl culture medium with 10% FBS was added in the lower chamber and used as a chemoattractant to encourage cell migration. For the invasion assay, the upper chamber of the transwell inserts were pre‐coated with 100 µl of 300 µg ml^−1^ Matrigel (Corning), and the cells were cultured as the migration assay. After 24 h incubation at 37 °C, all cells were stained with 0.1% crystal violet and the non‐migrated or non‐invaded cells were gently removed by cotton swab. The migrated or invaded cell were photographed and counted under an inverted microscope in five fields.

### Cell Proliferation Assay

Cell proliferation was assessed by the Cell Counting Kit‐8 (CCK‐8) (Yeasen) method. GBC cells in single‐cell suspension were seeded at 2000 cells/well into 96‐well plated with 100 µl culture medium. The 10 µl of CCK‐8 solution was added to the cells at the indicated time points and cells were incubated for 1 h at 37 °C. The reaction product was quantified at the absorbance at 450 nm using Synergy 2 microplate reader (Biotek).

### Western Blot

Western blot was performed using standard procedures. Total proteins were extracted from GBC cells using RIPA lysis buffer (Yeasen), and were quantified unsing a Micro BCA Protein Assay Kit (Thermo‐Fisher Scientific). 20 µg of total protein was electrophoresed through 10% SDS polyacrylamide gel and were then transferred to PVDF membranes (Millipore). The membranes were blocked in 5% skim milk for 1 h at room temperature and then incubated with primary antibodies at 4 °C overnight. Secondary antibodies were labeled with HRP, and the signals were detected using ECL Kit (Millipore) by the ChemiDoc XRS+ imaging System (Bio‐Rad). A β‐actin antibody was used as a control for whole‐cell lysates. The relative protein expression was calculated by Image J software and normalized to the expression of β‐actin. The antibody against DNMT3A (#32 578, dilution 1:1000) and p‐YAP (#13 008, dilution 1:1000) were purchased from Cell Signaling Tech, β‐actin (A1978, dilution 1:10 000), Flag (SAB1306078, dilution 1:10 000), and HA (H6908, dilution 1:10 000) were purchased from Sigma–Aldrich, CDH1 (ab40772, dilution 1:5000) and N‐cadherin (ab76011, dilution 1:5000) were purchased from Abcam, YAP (13584‐1‐AP, dilution 1:2000) and TAZ (23306‐1‐AP, dilution 1:1000) were purchased from Proteintech.

### RNA Extraction and Real‐Time Quantitative PCR

Total RNA was extracted from GBC cells or tissues using TRI Reagent (Sigma–Aldrich) following the manufacturer's protocol, and 1 µg of total RNA was reverse transcribed using the 1st Strand cDNA Synthesis SuperMix (Yeasen) into cDNA. Real‐time quantitative PCR (RT‐qPCR) was performed in triplicates on an Applied Biosystems ViiA 7 Real‐Time PCR system (Applied Biosystem). The relative mRNA expression was calculated with 2^−ΔΔCt^ method and normalized to internal reference gene ACTB. The primers for real‐time PCR as follows: DNMT3A, forward 5′‐GCCTCAATGTTACCCTGGAA‐3′, reverse 5′‐CAGCAGATGGTGCAGTAGGA‐3′; CDH1, forward 5′‐GAACGCATTGCCACATACAC‐3′, reverse 5′‐ATTCGGGCTTGTTGTCATTC‐3′; YAP, forward 5′‐CACAGCATGTTCGAGCTCAT‐3′, reverse 5′‐GATGCTGAGCTGTGGGTGTA‐3′; TAZ, forward 5′‐CATGGCAAGACCCTAGGAAG‐3′, reverse 5′‐TGCTGGTGTTGGTGATTCAT‐3′; AXL, forward 5′‐TGGCTGTGAAGACGATGAAG‐3′, reverse 5′‐TCGTTCAGAACCCTGGAAAC‐3′; KLF4, forward 5′‐GTCTCTTCGTGCACCCACTT‐3′, reverse 5′‐TGCTCAGCACTTCCTCAAGA‐3′; OCT4, forward 5′‐AGCGATCAAGCAGCGACTAT‐3′, reverse 5′‐GTGAAGTGAGGGCTCCCATA‐3′; MYC, forward 5′‐TCAAGAGGCGAACACACAAC‐3′, reverse 5′‐TAACTACCTTGGGGGCCTTT‐3′; CTCF, forward 5′‐GTGTTCCATGTGCGATTACG‐3′, reverse 5′‐TCATGTGCCTTTTCAGCTTG‐3′; ACTB, forward 5′‐CATGTACGTTGCTATCCAGGC‐3′, reverse 5′‐CTCCTTAATGTCACGCACGAT −3′.

### Tumor Metastasis‐Related Genes PCR Array

The total RNA (1 µg) was reverse transcribed into cDNA using the 1st Strand cDNA Synthesis SuperMix (Yeasen). The PCR array profiling of 84 tumor metastasis related genes and five housekeeping genes in 96 wells (LabEx) were detected by Applied Biosystems ViiA 7 Real‐Time PCR system (Applied Biosystem).

### Bisulfite Sequencing PCR (BSP) and Methylation‐Specific Quantitative PCR (MS‐qPCR) Assays

Genomic DNA was extracted from GBC cells by using the QIAamp DNA blood mini kit (Qiagen), and bisulfite treatment was performed by using the DNA Methylation‐Gold Kit (Zymo Research), following the manufacturer's introduction. For BSP assays, modified DNA was amplified, and PCR products were gel‐purified and sub‐cloned into a pESI‐T vector system (Yeasen). Ten colonies were sequenced to assess the degree of methylation and each CpG site by QUMA.^[^ ^33^ ^]^ For MS‐qPCR assays, the modified DNA was amplified to determine the methylation status of the promoter region of target gene as described previously.^[^ ^34^ ^]^ The primers were used for BSP as follow: CDH1, forward 5′‐TTTAGTAATTTTAGGTTAGAGGGTTAT‐3′, reverse 5′‐AAACTCACAAATACTTTACAATTCC‐3′; and the primers were used for MS‐qPCR as follows: CDH1, forward 5′‐TAATTTTAGGTTAGAGGGTTATCGC‐3′, reverse 5′‐CTCACAAATACTTTACAATTCCGAC‐3′, and ACTB, forward 5′‐TGGTGATGGAGGAGGTTTAGTAAGT‐3′, reverse 5′‐AACCAATAAAACCTACTCCTCCCTTAA‐3′

### Luciferase Assay

The promoter sequences (−500 to +191 bp) of CDH1 genome were cloned into pGL3‐basic promoter vector. Luciferase assay were performed in HEK293T cells with pGL3‐CDH1 promoter wild‐type luciferase reporter or YAP/TAZ‐TEADs binding motif mutant luciferase reporter. 500 ng reporter plasmids, 50 ng pRL‐TK‐Renilla‐luciferase plasmid which used for normalizing the transfection efficiency, together with 500 ng required overexpressed plasmids or empty vectors, were co‐transfected. 24 h post‐transfection, cells were lysed and the Firefly and Renilla luciferase activities were determined according to the manuscript's protocol of Dual‐Luciferase Reporter Assay System (Promega). The CDH1 promoter activity was calculated by the ratio of Firefly to Renilla.

### In Vitro Methylation Assay

The reporter plasmid of pGL3‐CDH1 was methylated by M.SssI (New England Biolabs) and SAM or mock methylated in the absence of M.SssI and SAM for 1 h at 37 °C. The vitro methylated efficiency was detected by HpaII (New England Biolabs) digestion that the totally methylated plasmids would not be digested into small fragments by HpaII. After that, the in vitro methylated reporter plasmids were transfected to HEK293T cells and perform dual‐luciferase reporter assays.

### Co‐Immunoprecipitation

For Co‐immunoprecipitation (Co‐IP) assay, HEK293T cells were transfected with indicated plasmids for 48 h, and then cells were lysed in IP lysis buffer containing protease inhibitor cocktail (Sigma–Aldrich) and PMSF (Sigma–Aldrich). The lysates were collected and immunoprecipitated with Flag or HA primary antibody at 4 °C overnight followed by incubated with Protein A agarose (Sigma‐Aldrich) at 4 °C for 2 h. The immunocomplexes were washed with IP lysis buffer, then eluted in 1×SDS loading buffer and subjected to western blot analysis. The primary antibodies of Flag (MAB3118) and HA (H6908) for immunoprecipitation were both purchased from Sigma–Aldrich.

### Immunofluorescence (IF)

GBC cells were plated in 6‐well plates covered with sterile coverslips. After fixed with ice‐cold 100% methanol at −20 °C for 10 min, cells were blocked in Blocking Buffer (5% normal serum, 0.3% Triton X‐100 in PBS) at room temperature for 1 h. Then incubating with primary antibody at 4 °C overnight, and incubated in fluorochrome‐conjugated secondary antibody diluted in Antibody Dilution Buffer (1% BSA, 0.3% Triton X‐100 in PBS) at room temperature in dark for 1 h. Nuclei were stained with 4,6‐diamidino‐2‐phenylindole (DAPI). Antibody against DNMT3A (HPA026588, dilution 1:100) was purchased from Sigma–Aldrich, and YAP (13584‐1‐AP, dilution 1:50) was purchased from Proteintech.

### Chromatin Immunoprecipitation Assay

Chromatin immunoprecipitation (ChIP) assay was performed using SimpleChIP Enzymatic Chromatin IP Kit (Cell Signaling Tech) according to the manufacturer's guidance. Briefly, the enzymatic digested chromatin was immunoprecipitated with primary antibody of DNMT3A (#32 578, Cell Signaling Tech), 5mC (SAB2702243, Sigma–Aldrich), YAP (#14 074, Cell Signaling Tech), or IgG (#2729, Cell Signaling Tech) overnight at 4 °C. The antibody/chromatin complexes were incubated with Protein G agarose for 2 h at 4 °C, finally eluted and purified. For ChIP‐qPCR assay, the immunoprecipitified DNA was quantified by qPCR method. For ChIP‐PCR assay, the immunoprecipitified DNA was quantified by PCR and loaded in Agarose gel for imaging. The primer used for anti‐DNMT3A or anti‐5mC ChIP on CDH1 promoter as following: forward 5′‐TAGAGGGTCACCGCGTCTAT‐3′, reverse 5′‐CTGATTGGCTGAGGGTTCAC‐3′. The primer used for anti‐YAP ChIP on CDH1 promoter as following: forward 5′‐CCCTTTCTGATCCCAGGTCT‐3′, reverse 5′‐GCCTGGAGTTGCTAGGGTCT‐3′.

### Liver Metastasis Xenografts Model

DNMT3A‐silenced or DNMT3A‐overexpressed and parental control GBC cells (2×10^6^) were subcutaneously injected into the right lower regions of 4‐week‐old male BALB/c nude mice, and then the mice were housed in laminar flow cabinets under specific pathogen conditions with food and water provided ad libitum. The mice were sacrificed at the 6th weeks, and livers were dissected and made into sections for hematoxylin and eosin (H&E) staining. In vivo studies were conducted in accordance with the National Institutes of Health Guidelines for the Care and Use of Laboratory Animals, and the study procedures were approved by the Institutional Animal Care and Use Committee of Renji Hospital affiliated to Shanghai Jiao Tong University School of Medicine.

### Clinical Samples

A total of 128 GBC tissues were collected from the Department of Biliary‐Pancreatic Surgery at Renji hospital. The definition of overall survival (OS) was the interval between the date of surgery and last follow‐up or death. The Ethics Committees of Renji Hospital affiliated to Shanghai Jiao Tong University School of Medicine approved the study protocols, and written informed consent was obtained from all subjects in this study. All the research was carried out in accordance with the provisions of the Helsinki Declaration of 1975.

### Immunohistochemistry Staining Analysis

Immunohistochemistry (IHC) staining was performed as described in our previous reported.^[^ ^8^ ^]^ Briefly, the GBC tumor samples were stained with DNMT3A (HPA026588, dilution 1:200, Sigma–Aldrich), YAP (13584‐1‐AP, dilution: 1:100, Proteintech), and CDH1 (SAB2701861, dilution 1:100, Sigma–Aldrich), respectively. The staining (defined as histoscore) was scored as the intensity of positive staining (0 = none; 1 = weak; 2 = moderate; 3 = strong) multiplied with the proportion of positive staining (0 = none; 1 = 1%–10%; 2 = 11%–50%; 3 = 51%–80%; 4 = 81%–100%). Sample with histoscore of more than 4 were considered to be high, and less than 4 were considered to be low. These scores were independently determined by two pathologists.

### Statistical Analysis

All data in this study were obtained from three independent experiments and presented as mean ± SEM. An unpaired Student's *t* test and Chi‐square test was used to analyze the group comparison of normally distributed measurement data and categorical data, respectively. The Kapan‐Meier method and log‐rank test were used to estimate the survival probabilities. Cox proportional hazards regression model was performed in univariate and multivariate analysis to determine the prognostic factors for GBC patients. Pearson correlation coefficient were used to analyzed the correlation between DNMT3A, YAP, and CDH1 protein expression. The differences were considered statistically significant if *P* < 0.05, and indicated by, ^*^ *P* < 0.05, ^**^ *P* < 0.01, ^***^ *P* < 0.001; or ns, not significant.

## Conflict of Interest

The authors declare no conflict of interest.

## Author Contributions

S.X. and Z.Y. contributed equally to this work. S.X., Q.L., and T.C. participated in research design; Q.L. and T.C. supervised the study; S.X. and Z.Y. conducted experiments and performed the experiments with the assistance from C.J. and W.C. S.X. wrote the manuscript, and T.C. revised the paper.

## Supporting information

Supporting Information

## Data Availability

The data that support the findings of this study are available from the corresponding author upon reasonable request.
