# Supplementary material for: DNMT3A Cooperates with YAP/TAZ to Drive Gallbladder Cancer Metastasis
Source: Adv Sci (Weinh). 2024 Feb 21;11(16):2308531. doi: 10.1002/advs.202308531 (PMC11040361; doi:10.1002/advs.202308531)
Supplement: Supplementary file 1 — Supporting Information [file ADVS-11-2308531-s001.pdf]

ADVANCED SCIENCE
Open Access

Supporting Information

for *Adv. Sci.*, DOI 10.1002/advs.202308531

DNMT3A Cooperates with YAP/TAZ to Drive Gallbladder Cancer Metastasis

Sunwang Xu, Zhiqing Yuan, Cen Jiang, Wei Chen, Qiwei Li and Tao Chen**

Supporting Information for

DNMT3A Cooperates with YAP/TAZ to Drive Gallbladder Cancer Metastasis

Sunwang Xu, Zhiqing Yuan, Cen Jiang, Wei Chen, Qiwei Li[*]*, Tao Chen*[*]

Contents:

7 Supplementary Figures and Legends.

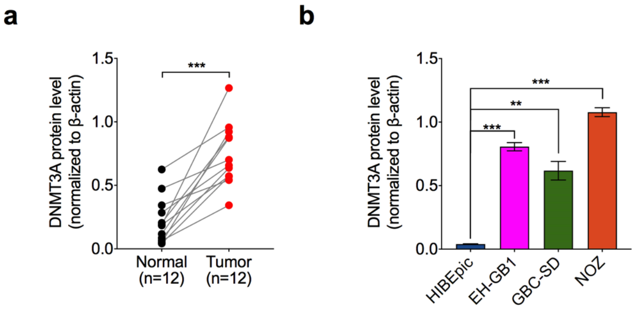

Figure S1. DNMT3A expression in GBC tissues and cells, related to Figure 1. a) Statistical analysis of DNMT3A protein levels in 12 representative paired GBC tumorous tissues and adjacent normal tissues, related to Figure 1a. Paired Student's *t* test, $^{***}P$<0.001. b) Statistical analysis of DNMT3A protein levels in normal human intrahepatic biliary epithelial cell (HIBEpiC) and three GBC cell lines (EH-GB1, GBC-SD, and NOZ) (n=3), related to Figure 1c. Unpaired Student's *t* test, $^{**}P$<0.01, $^{***}P$<0.001.

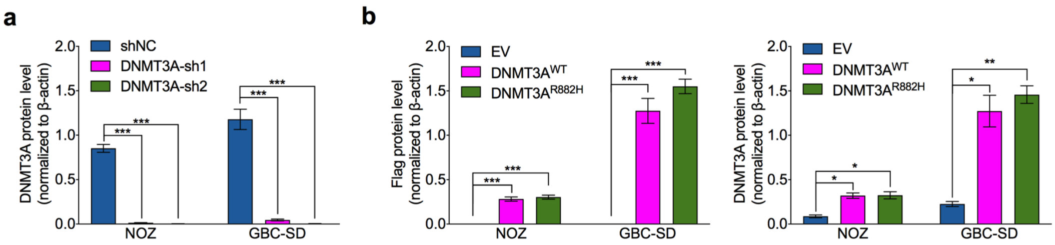

Figure S2. DNMT3A expression in GBC cells with DNMT3A knockdown or overexpression, related to Figure 2. a) Statistical analysis of DNMT3A protein levels in NOZ and GBC-SD cells (n=3) stably transfected with DNMT3A-shRNAs (DNMT3A-sh) or control shRNA (shNC), related to Figure 2a. Unpaired Student's *t* test, $^{***}P$<0.001. b) Statistical analysis of Flag-tag and DNMT3A protein levels in NOZ and GBC-SD cells (n=3) stably transfected with DNMT3A wild type construct (DNMT3A^{WT}), catalytic mutation construct (DNMT3A^{R882H}), or empty vector (EV), related to Figure 2e. Unpaired Student's *t* test, $^{*}P$<0.05, $^{**}P$<0.01.

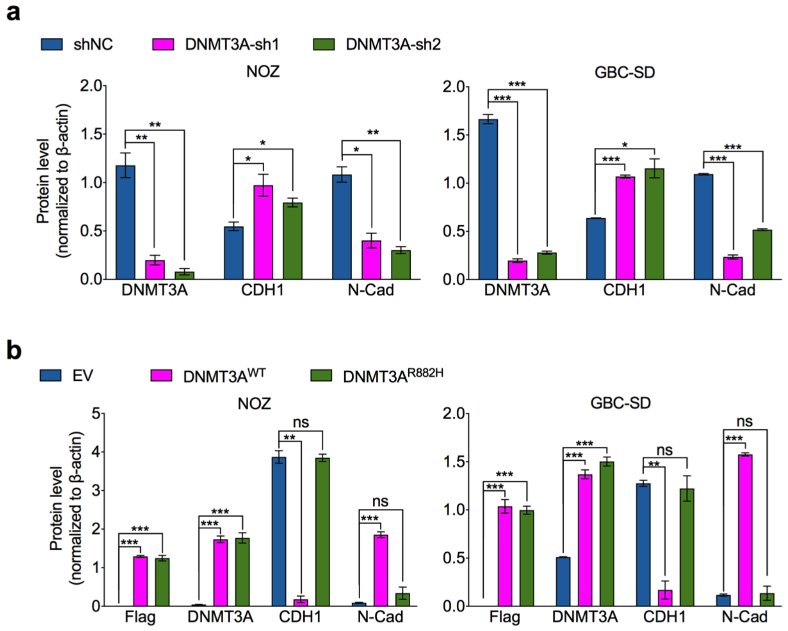

Figure S3. Statistical analysis for western blot results in GBC cells with DNMT3A knockdown or overexpression, related to Figure 4. a) Statistical analysis of DNMT3A, CDH1, and N-cadherin (N-Cad) protein levels in DNMT3A-depleted and control NOZ and GBC-SD cells (n=3), related to Figure 4b. Unpaired Student's *t* test, $^{*}P<0.05$, $^{**}P<0.01$, $^{***}P<0.001$. b) Statistical analysis of Flag-tag, DNMT3A, CDH1, and N-Cad protein levels in $DNMT3A^{WT}$, $DNMT3A^{R882H}$, or empty EV ectopic expressed NOZ and GBC-SD cells (n=3), related to Figure 4d. Unpaired Student's *t* test, $^{**}P<0.01$, $^{***}P<0.001$, ns, not significant.

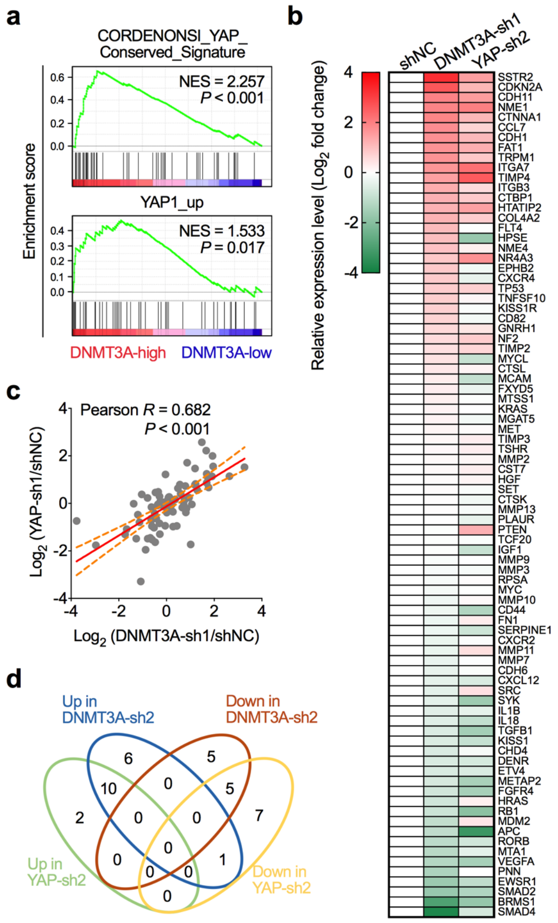

Figure S4. Co-regulated genes in DNMT3A knockdown and YAP knockdown GBC cells, related to Figure 5. a) GSEA results showed the YAP conserved signature was enriched in DNMT3A high expressed GBC tissues. b) A PCR array based analysis to detect the expression levels of 84 tumor metastasis related genes' expression in DNMT3A knockdown, YAP knockdown, and control NOZ cells. The heatmap showed the expression changes of all genes in DNMT3A knockdown and YAP knockdown NOZ cells by comparing to control NOZ cells. c) Comparison of the expression changes of all 84 tumor metastasis related gene between DNMT3A-knockdown and YAP-knockdown NOZ cells. d) Venn diagram shows the co-upregulated or co-downregulated genes in DNMT3A-knockdown and YAP-knockdown NOZ cells.

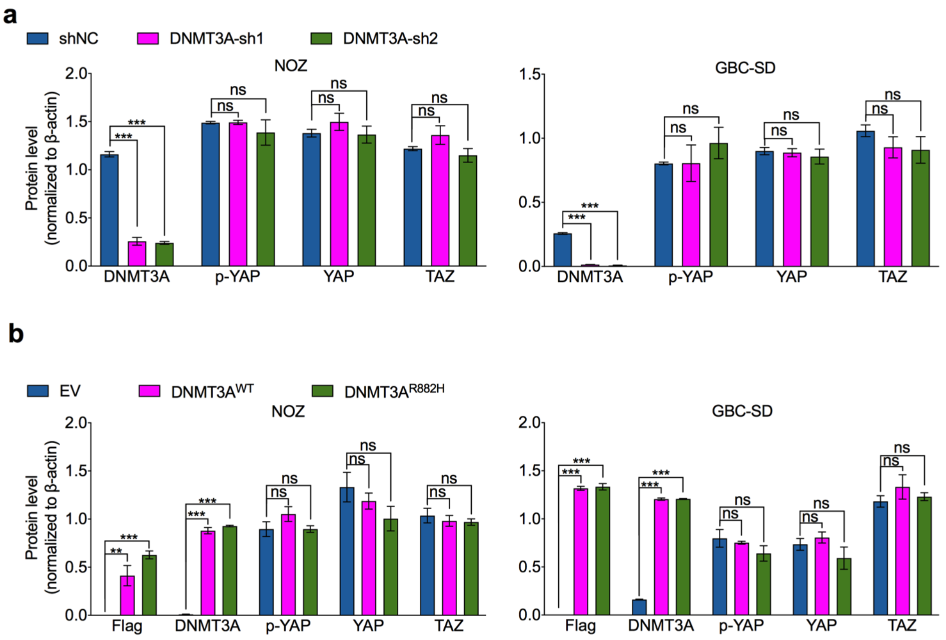

Figure S5. Statistical analysis for western blot results in GBC cells with DNMT3A knockdown or overexpression, related to Figure 5. a) Statistical analysis of DNMT3A, Ser127-phosphorylated YAP (p-YAP), YAP, and TAZ protein levels in DNMT3A-depleted and control NOZ and GBC-SD cells (n=3), related to Figure 5c. Unpaired Student's *t* test, $^{***}P<0.001$, ns, not significant. (b) Statistical analysis of Flag-tag, DNMT3A, p-YAP, YAP, and TAZ protein levels in $DNMT3A^{WT}$, $DNMT3A^{R882H}$, or empty EV ectopic expressed NOZ and GBC-SD cells (n=3), related to Figure 5d. Unpaired Student's *t* test, $^{**}P<0.01$, $^{***}P<0.001$, ns, not significant.

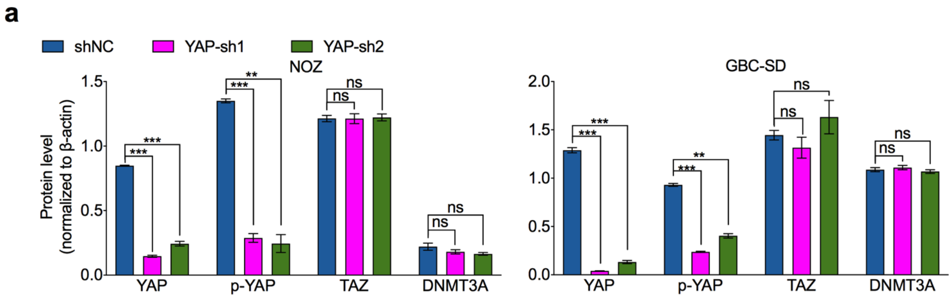

Figure S6. Statistical analysis for western blot results in GBC cells with YAP knockdown, related to Figure 6. (a) Statistical analysis of YAP, p-YAP, TAZ, and DNMT3A protein levels in NOZ and GBC-SD cells (n=3) stably transfected with YAP-shRNAs (YAP-sh) or control shRNA (shNC), related to Figure 6a. Unpaired Student's *t* test, $^{**}P<0.01$, $^{***}P<0.001$, ns, not significant.

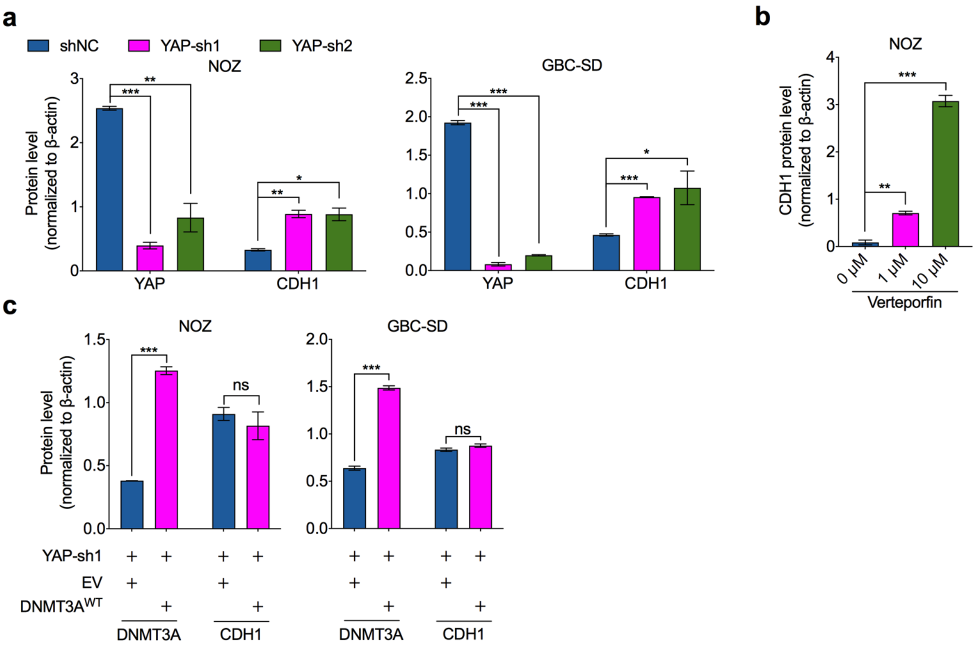

Figure S7. Statistical analysis for western blot results in GBC cells, related to Figure 7. a) Statistical analysis of YAP and CDH1 protein levels in DNMT3A-depleted and control NOZ and GBC-SD cells (n=3), related to Figure 7a. Unpaired Student's *t* test, $^{*}P$<0.05, $^{**}P$<0.01, $^{***}P$<0.001. b) Statistical analysis of CDH1 protein levels in NOZ cells treated with concentration-gradient of verteporfin (n=3), related to Figure 7f. Unpaired Student's *t* test, $^{**}P$<0.01, $^{***}P$<0.001. c) Statistical analysis of DNMT3A and CDH1 protein levels in DNMT3A^{WT} or EV transfected YAP-depleted NOZ and GBC-SD cells (n=3), related to Figure 7k. Unpaired Student's *t* test, $^{***}P$<0.001, ns, not significant.
